# Pathogenic Mannheimia haemolytica Invades Differentiated Bovine Airway Epithelial Cells

**DOI:** 10.1128/IAI.00078-19

**Published:** 2019-05-21

**Authors:** Daniel Cozens, Erin Sutherland, Miquel Lauder, Geraldine Taylor, Catherine C. Berry, Robert L. Davies

**Affiliations:** a Institute of Infection, Immunity and Inflammation, College of Medical, Veterinary and Life Sciences, University of Glasgow, Glasgow, United Kingdom; b The Pirbright Institute, Pirbright, Surrey, United Kingdom; c Institute of Molecular, Cell and Systems Biology, College of Medical, Veterinary and Life Sciences, University of Glasgow, Glasgow, United Kingdom; Washington State University

**Keywords:** Mannheimia haemolytica, bacterial pathogenesis, bovine respiratory disease, host-pathogen interactions

## Abstract

The Gram-negative bacterium Mannheimia haemolytica is the primary bacterial species associated with bovine respiratory disease (BRD) and is responsible for significant economic losses to livestock industries worldwide. Healthy cattle are frequently colonized by commensal serotype A2 strains, but disease is usually caused by pathogenic strains of serotype A1. For reasons that are poorly understood, a transition occurs within the respiratory tract and a sudden explosive proliferation of serotype A1 bacteria leads to the onset of pneumonic disease. Very little is known about the interactions of M. haemolytica with airway epithelial cells of the respiratory mucosa which might explain the different abilities of serotype A1 and A2 strains to cause disease. In the present study, host-pathogen interactions in the bovine respiratory tract were mimicked using a novel differentiated bovine bronchial epithelial cell (BBEC) infection model. In this model, differentiated BBECs were inoculated with serotype A1 or A2 strains of M. haemolytica and the course of infection followed over a 5-day period by microscopic assessment and measurement of key proinflammatory mediators. We have demonstrated that serotype A1, but not A2, M. haemolytica invades differentiated BBECs by transcytosis and subsequently undergoes rapid intracellular replication before spreading to adjacent cells and causing extensive cellular damage. Our findings suggest that the explosive proliferation of serotype A1 M. haemolytica that occurs within the bovine respiratory tract prior to the onset of pneumonic disease is potentially due to bacterial invasion of, and rapid proliferation within, the mucosal epithelium. The discovery of this previously unrecognized mechanism of pathogenesis is important because it will allow the serotype A1-specific virulence determinants responsible for invasion to be identified and thereby provide opportunities for the development of new strategies for combatting BRD aimed at preventing early colonization and infection of the bovine respiratory tract.

## Abstract (precis)

The Gram-negative bacterium Mannheimia haemolytica is the primary bacterial species associated with bovine respiratory disease (BRD) and is responsible for significant economic losses to livestock industries worldwide. Healthy cattle are frequently colonized by commensal serotype A2 strains, but disease is usually caused by pathogenic strains of serotype A1.

## INTRODUCTION

Mannheimia haemolytica is a Gram-negative bacterium and is the primary bacterial species associated with bovine respiratory disease (BRD), a multifactorial condition of cattle involving poorly understood interactions between various bacterial and viral pathogens and the host (1–3). Bovine respiratory disease is responsible for significant economic losses (>$1 billion to $3 billion annually in the United States alone) to the livestock industries worldwide (1, 4–6). Antibiotics play an important role in the control of BRD, but the incidence of multidrug-resistant bacterial strains is increasing (7–12), and there are serious public health concerns associated with the increased use of antimicrobial drugs in food-producing animals (1, 3, 13–15). Therefore, alternative, less extensively drug-dependent strategies are required to control disease. Vaccination is widely used for the prevention of BRD, but the efficacy of currently available vaccines is inconsistent, and improved vaccines are required (15, 16). However, the development of improved vaccines and other control measures is hindered by our limited understanding of the pathogenesis of BRD.

Mannheimia haemolytica occurs naturally as a commensal in the upper respiratory tract (URT) of healthy cattle but, under circumstances which are poorly understood, is frequently associated with disease (3, 5, 17). The bacterium comprises 12 capsular serotypes (18). Healthy cattle are often colonized by commensal strains of serotype A2, but disease is almost always caused by pathogenic isolates of serotype A1 (1, 3, 5, 6, 9, 14). For reasons that are unclear but that are associated with crowding, stress, and/or viral infection, a sudden explosive proliferation occurs in the number of serotype A1 bacteria present in the URT of susceptible animals (5, 6, 17, 19). The colonization of the mucosal surfaces leads to inhalation of bacterium-containing aerosol droplets into the lungs and predisposes the animals to the onset of pneumonic disease (20, 21). Thus, pneumonia appears to be the consequence of two events—the first occurring in the URT and the second in the lungs (19).

Events within the lungs are relatively well defined. The secretion of leukotoxin and the release of lipopolysaccharide together play a central role in the migration of neutrophils into the lungs, and these immune cells are largely responsible for the excessive pulmonary inflammation and tissue damage associated with BRD (5, 6, 22–24). In contrast, the reasons for the very different behaviors of serotype A1 and A2 strains within the URT during the early stages of colonization, and, indeed, the reasons for their differing abilities to cause disease, are not known. Serotype A1 and A2 strains of M. haemolytica differ in a wide range of virulence-associated characteristics (25–31), but there is little clear-cut evidence that any of them have specific roles which might explain unequivocally the differences in the levels of pathogenicity of these strains. Due partly to the lack of availability of physiologically relevant and reproducible *in vitro* methodologies, there has been very little focus on improving our understanding of the early interactions of M. haemolytica with the respiratory epithelium prior to the onset of disease. We believe that understanding these early host-pathogen interactions is key to explaining the differential responses of serotype A1 versus A2 M. haemolytica strains with respect to high-level nasopharyngeal colonization and/or disease causation.

Airway epithelial cells (AECs) play important roles in defense of the respiratory tract. The respiratory epithelium provides a physicochemical barrier against inhaled microorganisms and particulates which involves the presence of intercellular junctions and mucociliary clearance (32, 33). Furthermore, AECs are involved in the innate immune response and, during BRD, are the source of the proinflammatory mediators which stimulate the activation and regulation of neutrophils and macrophages (22, 34, 35). Submerged AEC cultures, including either primary cells or immortalized cell lines, have been used to investigate interactions of M. haemolytica (34, 36–38) with the bovine respiratory tract, but these have various limitations: the cultures do not reflect the multicellular complexity of the parental tissue *in vivo* and lack its three-dimensional (3-D) architecture, and the physiological conditions are not representative of those found within the bovine URT. However, those characteristics that are lacking in submerged cultures can be recapitulated using differentiated AECs grown at an air-liquid interface (ALI) and such cell culture approaches have been used to study the interactions of various bacterial and viral pathogens with different host species (39–57). Indeed, with a view to similarly studying the interactions of M. haemolytica with the bovine respiratory epithelium, we have established optimum culture conditions for the growth and differentiation of bovine bronchial epithelial cells (BBECs) grown at an ALI (58) and have identified a 21-to-42-day window during which these cultures are fully differentiated, healthy, and suitable for infection studies (59).

In the present study, we hypothesized that serotype A1 and A2 strains of M. haemolytica interact with and stimulate differentiated BBECs in different ways. In particular, since various bacterial pathogens of the human respiratory tract are known to invade human AECs (55, 56, 60–63), we wished to explore the possibility that serotype A1 M. haemolytica invades bovine AECs. Here, we demonstrated that pathogenic serotype A1 M. haemolytica, but not commensal serotype A2 M. haemolytica, invades and replicates within bovine AECs. Importantly, the discovery of this invasion process provides a possible explanation for the explosive proliferation of serotype A1 bacteria that occurs in the bovine URT before the onset of pneumonic disease and thereby opens avenues for the development of new disease intervention strategies.

## RESULTS

### Mannheimia haemolytica serotype A1 but not serotype A2 colonizes differentiated BBECs.

**FIG 1:**
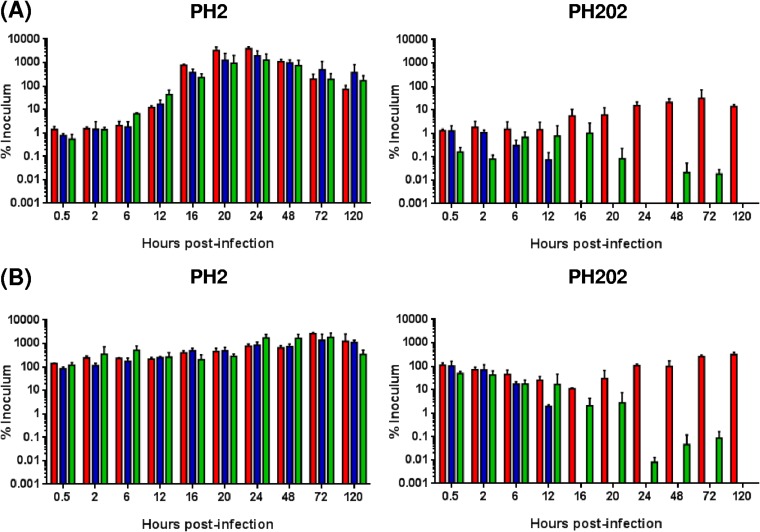
Bacterial enumeration of differentiated BBEC cultures infected with M. haemolytica isolates PH2 and PH202 over a 5-day time course. Differentiated BBEC cultures were infected with M. haemolytica isolates PH2 and PH202 (2.5 × 10^7^ CFU/insert) at day 21 post-ALI and maintained for 5 days. Adherence and colonization of (A) bacteria associated with the epithelial cells after apical washing and (B) bacteria present in the apical washes were assessed by enumeration at the indicated time points. The bacteria were enumerated by counting viable cells, and the numbers are expressed as percentages of the original inoculum. Three cultures were analyzed per time point, and the data represent means ± standard deviations of results from cultures derived from three different animals (red columns, animal 1; blue columns, animal 2; green columns, animal 3).

The ability of M. haemolytica serotype A1 (PH2) and serotype A2 (PH202) isolates to adhere to and colonize differentiated BBECs was first assessed by bacterial enumeration at selected time points from 0.5 to 120 h postinfection (p.i.) (Fig. 1). The levels of adherence of the two isolates were similar at very early time points (0.5 and 2 h p.i.). A relatively small number of bacteria, representing ∼1% of the inoculum, adhered to the BBECs at each time point (Fig. 1A); in contrast, a very high number of bacteria, comprising the majority of the inoculum, was present in the apical washes at each time point (Fig. 1B). However, the fates of the bacteria representing each of the two isolates associated with the BBECs were subsequently very different. From 6 h p.i., the bacterial numbers representing serotype A1 isolate PH2 increased exponentially and achieved a maximum (>1,000% of the inoculum) at 24 h p.i.; thereafter, the numbers declined to ∼100% of the inoculum by 120 h (Fig. 1A). The number of serotype A1 bacteria within the washes increased marginally at the same time points (Fig. 1B), but this increase did not match the exponential increase observed for the bacteria associated with the BBECs between 6 and 24 h (Fig. 1A). These results suggest that a high proportion of the increased bacterial numbers observed in Fig. 1A (at between 6 and 24 h) remained associated with the tissue and were not removed by washing. In contrast, the numbers of serotype A2 isolate PH202 bacteria, including both those associated with the BBECs (Fig. 1A) and those present in the washes (Fig. 1B), either decreased (animals 2 and 3) or increased marginally (animal 1) from 6 h onwards. Thus, serotype A2 bacteria were completely cleared by 16 h from the animal 2 cultures, were cleared by 120 h from the animal 3 cultures, and were present in low numbers (∼10% of the inoculum) after 120 h in the animal 1 cultures. Taken together, these results clearly demonstrate marked differences in the ability of the M. haemolytica isolates to colonize differentiated BBEC cultures; the serotype A1 isolate was able to rapidly colonize the epithelial layer, whereas the serotype A2 isolate was unable to do so.

### Mannheimia haemolytica invades differentiated BBECs and forms foci of infection.

**FIG 2:**
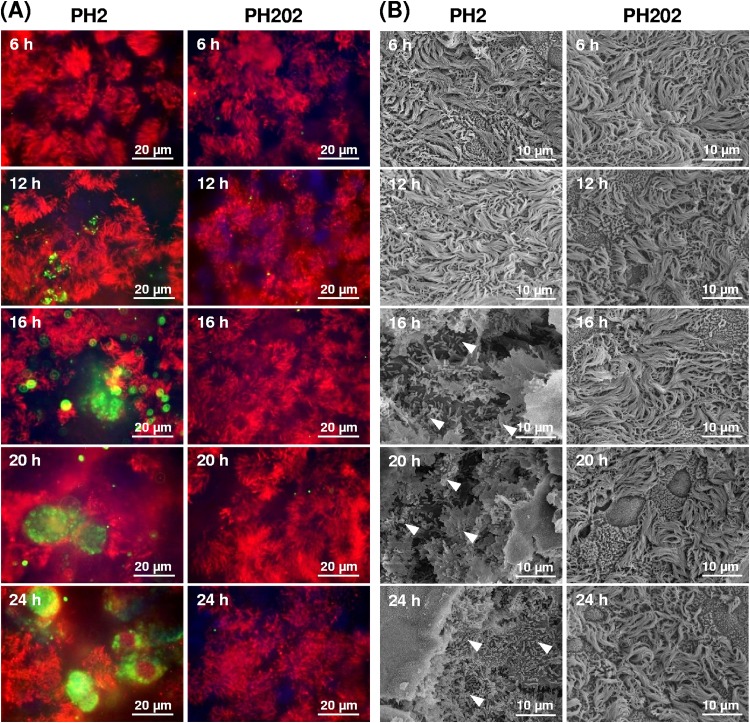
Microscopic analysis of differentiated BBEC cultures infected with M. haemolytica isolates PH2 and PH202 over a 5-day time course. Differentiated BBEC cultures were infected with M. haemolytica isolates PH2 and PH202 (2.5 × 10^7^ CFU/insert) at day 21 post-ALI and maintained for 5 days. At the indicated time points p.i., the cultures were washed to remove unbound bacteria and fixed. Bacterial colonization was subsequently assessed using (A) IFM and (B) SEM. As shown in panel A, increasing numbers of PH2 bacteria but not PH202 bacteria were associated with BBECs over time (bacteria, green; cilia [β-tubulin], red; nuclei, blue); as shown in panel B, increasing numbers of PH2 bacteria (arrowheads) but not PH202 bacteria were associated with damaged tissue from 16 h p.i. Data corresponding to further time points are shown in Fig. S1 and S2.

**FIG 3:**
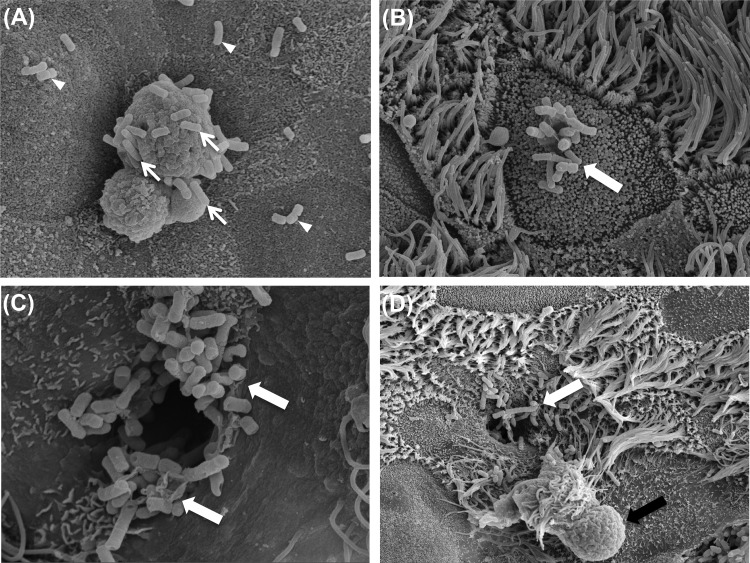
Scanning electron microscopy of differentiated BBEC cultures infected with M. haemolytica isolate PH2. Differentiated BBEC cultures were infected with M. haemolytica isolate PH2 (2.5 × 10^7^ CFU/insert) at day 21 post-ALI and maintained for 5 days at day 21 post-ALI and maintained for 5 days. At selected time points p.i., the cultures were washed to remove unbound bacteria, fixed, and examined by SEM. (A) Bacteria adhering to nonciliated epithelial cells (arrowheads) and to mucus (arrows). (B) Bacteria (arrow) adhering to the center of a nonciliated epithelial cell but not to cilia. (C) Large numbers of bacteria (arrows) associated with an invagination of the cell membrane. (D) Bacteria (white arrow) associated with an invagination of the cell membrane, which may have been the result of mucus extrusion (black arrow).

Colonization of the differentiated BBEC cultures was next evaluated using immunofluorescence microscopy (IFM) and scanning electron microscopy (SEM). Immunofluorescence microscopy of infected cultures revealed very little evidence of adherence by either isolate over the first 6 h of infection (Fig. 2A; see also Fig. S1 in the supplemental material), and this observation was generally confirmed by SEM (Fig. 2B; see also Fig. S2). However, further scrutiny by SEM revealed low levels of sporadic bacterial adherence over the apical surface (Fig. 3). Thus, small numbers of serotype A1 bacteria (typically 2 to 3 per cell) were observed to adhere to patches of epithelial cells (Fig. 3A, arrowheads), as well as occasionally to mucus (Fig. 3A, arrows), at early time points. Notably, serotype A1 bacteria adhered to the apical surfaces of nonciliated cells but not to the cilia of ciliated cells (Fig. 3B, arrow).

As expected from the data representing the viable counts (Fig. 1), the outcomes of infection with the serotype A1 and A2 strains were very different after 6 h. Immunofluorescence microscopy demonstrated that the bacterial numbers of serotype A1 isolate PH2 progressively increased from 6 h p.i. through successive time points up to 24 h (Fig. 2A); these observations corresponded with the increasing numbers of bacteria recovered from the cultures between 6 and 24 h (Fig. 1A). However, the bacteria were not evenly distributed across the epithelial surface but were instead initially present in relatively small numbers of clusters, or foci of infection, that appeared to be associated with nonciliated regions (Fig. 2A). The number, size, and density of these foci increased between 12 and 24 h, and later time points were associated with progressively diminished staining of cilia that indicated increased damage and destruction of the epithelial layer; this was most clearly observed at 72 and 120 h (Fig. S1). The identification of well-established and extensive foci of infection from 16 h p.i. by IFM was confirmed by SEM (Fig. 2B). Moreover, SEM revealed that from 16 h p.i. onward, these infection foci were characterized by invasion and disruption of the epithelial layer; large numbers of bacteria were clearly visible within deeper regions of infected, fractured tissue (Fig. 2B, arrowheads). At later time points (i.e., from 48 h onward), SEM revealed severe destruction of the epithelial layer such that the underlying membrane was exposed (Fig. S2). In striking contrast to isolate PH2, serotype A2 isolate PH202 continued to exhibit few or no signs of adherence from 6 h p.i. and there was no evidence of the colonization and invasion that was characteristic of isolate PH2 by either IFM (Fig. 2A; see also Fig. S1) or SEM (Fig. 2B; see also Fig. S2).

**FIG 4:**
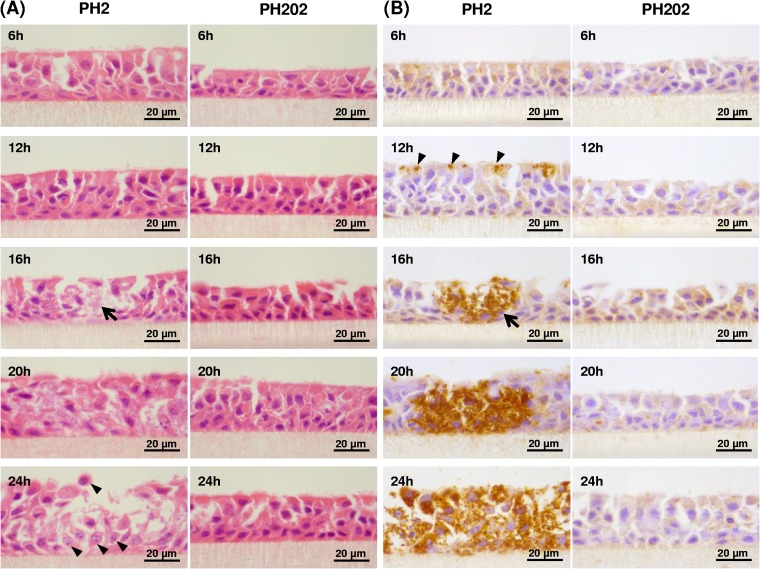
Histological analysis of differentiated BBEC cultures infected with M. haemolytica isolates PH2 and PH202 over a 5-day time course. Differentiated BBEC cultures were infected with M. haemolytica isolates PH2 and PH202 (2.5 × 10^7^ CFU/insert) at day 21 post-ALI and maintained for 5 days. At the indicated time points p.i., the cultures were washed to remove unbound bacteria, fixed, paraffin-embedded, and sectioned using standard histological techniques. Bacterial colonization and invasion were assessed using H&E staining (A) and IHC (B) (OmpA-labeled bacteria are stained brown). As shown in panel A, PH2 bacteria but not PH202 bacteria were observed within the epithelial layer from 16 h p.i. (arrow) and apoptotic and rounded cells were apparent at later time points (arrowheads). As shown in panel B, PH2 bacteria but not PH202 bacteria were identified within the epithelial layer from 12 h p.i. (arrowheads) and discrete foci of infection (arrow), penetrating the full depth of the epithelial layer, were visible by 16 h. Data from further time points are shown in Fig. S3 and S4.

Colonization of the differentiated BBEC cultures was also assessed by hematoxylin and eosin (H&E) and immunohistochemical (IHC) staining of histological sections. As expected, there was little evidence of bacterial adherence and colonization by either isolate over the first 6 h of infection but the fates of the bacteria representing each of the two isolates were again very different after 6 h (Fig. 4). At 12 h p.i., serotype A1 (PH2) bacteria were not discernible by H&E staining (Fig. 4A) but IHC staining revealed numerous small clusters of bacteria at the epithelial cell surface (Fig. 4B, arrowheads). By 16 h, bacteria were clearly observed within distinct foci of infection by H&E staining (Fig. 4A, arrow) and this was confirmed by IHC staining (Fig. 4B, arrow). Notably, this idea of a rapid increase in bacterial numbers between 12 and 16 h was supported by the IFM and SEM imaging (Fig. 2). These observations highlight that the 4-h period between 12 and 16 h p.i. represents a key transition stage during which infection rapidly progresses from the presence of small clusters of bacteria at the apical surface to extensive, deep-seated foci of infection which extend the full depth of the epithelial layer. Over the subsequent 8 h (16 to 24 h p.i.), the foci of infection became larger (due to the lateral spread of bacteria) and the bacteria more numerous (Fig. 4). Furthermore, from 16 h p.i., epithelial cells in the vicinity of the infection foci displayed cytopathic effects; large numbers of rounded and apoptotic cells were present (Fig. 4A, arrowheads). By 48 and 72 h, the integrity of the epithelial layer was significantly disrupted (Fig. S3 and S4), and epithelial fragments were removed during washing; these observations most likely account for the decline in bacterial numbers after 24 h described above (Fig. 1A). In contrast, and consistent with the IFM and SEM results, there was (with one exception) no evidence of bacterial colonization and tissue invasion by serotype A2 isolate PH202 (Fig. 4). Indeed, the epithelial layer remained intact until day 5 (Fig. S3 and S4) and maintained its ciliation and barrier function (described below). The single exception was the 120-h time point for animal 1, which showed some signs of bacterial colonization and tissue disruption (results not shown); this observation was in agreement with the recovery of low numbers of bacteria from cultures infected with isolate PH202 (Fig. 1A). A semiquantitative assessment of bacterial infection of the BBEC cultures from each of the three animals is shown in Table 1.

**TABLE 1:** Semiquantitative assessment of bacterial invasion of, and epithelial damage to, differentiated BBEC cultures infected with *M. haemolytica* isolates PH2 and PH202 over a 5-day time course^*a*^

Time postinfection (h)	Isolate PH2			Isolate PH202		
	Animal 1	Animal 2	Animal 3	Animal 1	Animal 2	Animal 3
0.5	−	−	−	−	−	−
2	−	−	−	−	−	−
6	−	−	−	−	−	−
12	+	+	−	−	−	−
16	++	++	++	−	−	−
20	++	++	++	−	−	−
24	++	++	++	−	−	−
48	+++	+++	+++	−	−	−
72	+++	+++	+++	−	−	−
120	+++	+++	+++	+++	−	−

a Differentiated BBEC cultures were infected with M. haemolytica isolates PH2 and PH202 (2.5 × 10^7^ CFU/insert) at day 21 post-ALI and maintained for five days. At the indicated time points p.i., sections of infected BBEC cultures were analyzed using H&E staining and IHC (see Fig. 4) (see also Fig. S3 and S4). Assessments of bacterial colonization/invasion and of the epithelial integrity of the histological sections were made semiquantitatively as follows: −, no evidence of infection, healthy epithelial layer; +, evidence of a minor degree of infection, a small number of foci of infection present, no tissue damage; ++, evidence of a moderate degree of infection, large numbers of foci of infection present, some epithelial damage; +++, evidence of a high degree of infection affecting the entire culture, extensive epithelial damage.

### Invasion of BBECs is independent of tight-junction integrity.

**FIG 5:**
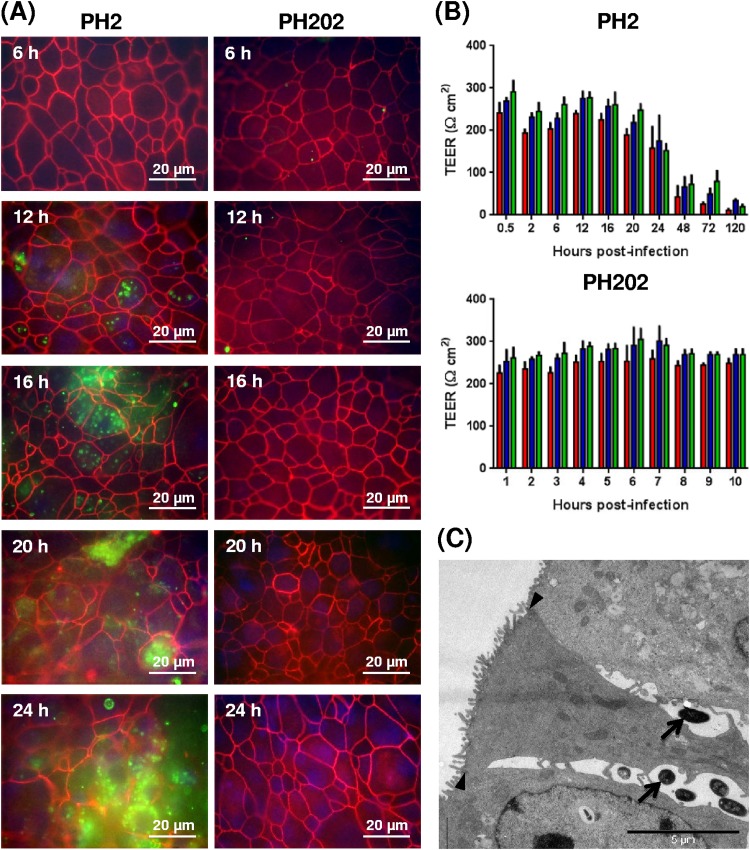
Tight-junction integrity of differentiated BBEC cultures infected with M. haemolytica isolates PH2 and PH202 over a 5-day time course. Differentiated BBEC cultures were infected with M. haemolytica isolates PH2 and PH202 (2.5 × 10^7^ CFU/insert) at day 21 post-ALI and maintained for 5 days. At the indicated time points p.i., the cultures were washed to remove unbound bacteria and fixed. Bacterial colonization and tight-junction integrity were subsequently assessed using IFM (bacteria, green; ZO-1, red; nuclei, blue) (A), TEER determination (B), and TEM (C). As shown in panel A, increasing numbers of PH2 bacteria but not PH202 bacteria were associated with BBECs over time and tight junctions remained intact until severe damage of epithelial cells occurred at later time points. As shown in panel B, infection with PH2 bacteria but not PH202 bacteria caused a rapid decline in tight-junction integrity (TEER) of BBEC cultures between 16 and 48 h p.i. In these experiments, three cultures were analyzed per condition, and data represent means ± standard deviations of results determined for cultures derived from three different animals (red columns, animal 1; blue columns, animal 2; green columns, animal 3). (C) TEM image of 24-h-PH2-infected BBECs showing bacteria (arrows) within the paracellular spaces between epithelial cells which possess intact tight junctions (arrowheads).

Tight junctions, together with adherens junctions and desmosomes, are involved in creating the hallmark barrier function of the respiratory tract and are specifically targeted and degraded by certain bacterial pathogens during paracellular infection processes (64–67). The tight-junction integrity of BBECs was assessed following challenge with isolates PH2 and PH202 by ZO-1 staining and transepithelial electrical resistance (TEER) determination. Normal tight-junction staining was observed within BBEC cultures infected with serotype A1 isolate PH2 at early time points (0.5 to 16 h p.i.); the integrity of the junctional complexes was unaffected by early M. haemolytica colonization (Fig. 5A; see also Fig. S5). In particular, the tight junctions of those epithelial cells which bacteria had most likely invaded at 12 and 16 h remained intact (Fig. S6A). However, as epithelial cells were damaged and disrupted at later time points (e.g., at 20 and 24 h), there was a simultaneous loss of tight-junction staining within infection foci, although the surrounding cells still maintained intact tight junctions (Fig. 5A). Confocal microscopy confirmed that tight junctions remained intact in areas adjacent to infection foci (Fig. S6B). At 48, 72, and 120 h, there was a loss of tight-junction staining over large areas of the epithelial layer which corresponded with the extensive tissue destruction described above (Fig. S5). These observations were reflected in the TEER measurements for isolate PH2 (Fig. 5B). The TEER values were maintained until 16 h p.i., but there was a significant reduction in the TEER values between 16 and 48 h p.i. (*P < *0.001; two-way analysis of variance [ANOVA]). Furthermore, transmission electron microscopy (TEM) of infected BBECs confirmed the presence of intact tight junctions between epithelial cells whose paracellular spaces contained numerous bacteria (Fig. 5C). In contrast, infection with serotype A2 isolate PH202 had no effect on tight-junction integrity over the 5-day time course as determined by ZO-1 staining (Fig. 5A; see also Fig. S5) and TEER measurement (Fig. 5B).

On the basis of the observations reported above, we hypothesized that serotype A1 isolate PH2 was not invading the epithelial layer by direct targeting of the tight junctions (i.e., via paracytosis) such as occurs in some bacterial pathogens (64–67). To test this hypothesis, we treated BBECs with lipoxin A_4_ (LXA_4_) prior to infection with PH2. Lipoxin A_4_ is a biologically active eicosanoid which stimulates tight-junction formation and repair in bronchial epithelial cells (68, 69). The TEER of BBEC cultures pre- and postinfection increased with increasing concentrations of LXA_4_ (Fig. S7A), confirming improved barrier function, but this was not accompanied by a reduction in the numbers of colonizing bacteria at 24 h p.i. (Fig. S7B) such as might be expected if bacteria were invading by this route.

### Mannheimia haemolytica invades differentiated BBECs by transcytosis and rapidly replicates intracellularly.

**FIG 6:**
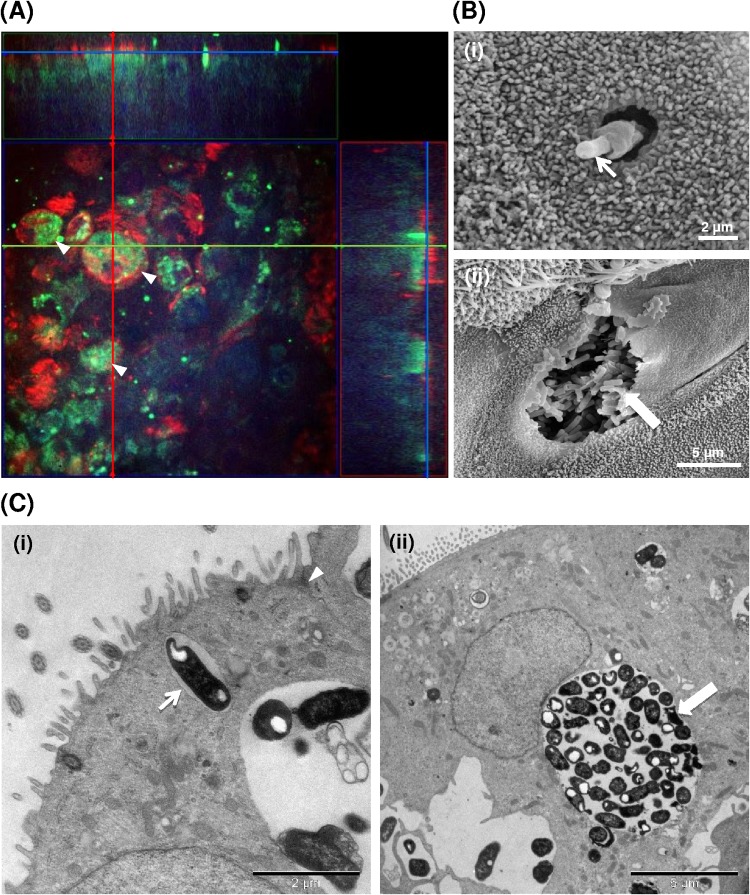
Internalization of M. haemolytica isolate PH2 by differentiated BBECs. Differentiated BBEC cultures were infected with M. haemolytica isolate PH2 (2.5 × 10^7^ CFU/insert) at day 21 post-ALI and maintained for 5 days. At selected time points p.i., the cultures were washed to remove unbound bacteria and fixed. Internalization of bacteria was subsequently assessed using IFM (bacteria, green; cilia [β-tubulin], red; nuclei, blue) (A), SEM (B), and TEM (C). (A) Z-stack orthogonal representation (×630 magnification) of a 24-h-infected BBEC culture showing bacteria located intracellularly within rounded epithelial cells (arrowheads). (B) Representative images of (i) potential bacterial uptake by a nonciliated epithelial cell (arrow) and (ii) an epithelial cell (with apical membrane partially removed) containing large numbers of internalized bacteria (arrow). (C) Representative images of (i) a single bacterium (arrow) within a small vesicle (endosome) in the cytoplasm of an infected cell (note intact tight junction [arrowhead]) and (ii) numerous bacteria within a larger vesicle (endosome) in the cytoplasm of an infected cell. Both images represent 20 h p.i.

The association of fluorescently labeled bacteria of serotype A1 isolate PH2 with the centers of infected BBECs, and their complete absence from cell peripheries (Fig. S6A, arrowheads), provided preliminary evidence that transcytosis, and not paracytosis, was the route of cellular invasion. Confocal microscopy was subsequently used to further identify the distribution of bacteria within infected BBEC cultures. Z-stack projections of infected cultures displayed high densities of bacteria confined within epithelial cell boundaries (Fig. 6A, arrowheads). The intracellular location of bacteria was confirmed using a gentamicin protection assay (Fig. S8). Following infection with isolate PH2, a small intracellular subpopulation of gentamicin-surviving bacteria was present at 12 h p.i., and the subpopulation had increased in number substantially by 24 h p.i. The number of intracellular bacteria had decreased by 48 h, a finding which is explained by the destruction and removal of the epithelium described above. Gentamicin-surviving (internalized) bacteria of serotype A2 isolate PH202 were not detected at any time points following challenge.

To address the key issue of how serotype A1 bacteria are internalized, more-extensive SEM and TEM analyses were performed. Potential evidence for bacterial uptake by epithelial cells was provided by SEM, although such events were observed infrequently; this may have been due to the polarization of the cells (51, 60) and/or to selective binding to a limited repertoire of cells (46). Large numbers of bacteria were occasionally observed to be closely associated with invaginations of the epithelial cell surface (Fig. 3C and D, white arrows). The close proximity of extruded mucus to some of these invaginations (Fig. 3D, black arrow) suggests that these epithelial cells were, in fact, goblet cells and that this cell type represents a potential portal of entry for M. haemolytica. There was also evidence of single bacterial cells being taken up by epithelial cells (Fig. 6B, panel i). However, evidence for the intracellular location and replication of serotype A1 bacteria was more readily obtained. Thus, large numbers of internalized bacteria were observed within epithelial cells by SEM (Fig. 6B, panel ii, arrow), suggesting that rapid intracellular replication occurs after uptake. Bacterial uptake and intracellular replication within BBECs were confirmed by TEM (Fig. 6C). Numerous membrane-bound vesicles containing bacteria were observed within BBECs infected with serotype A1 isolate PH2 at time points between 16 and 24 h p.i. Many vesicles contained only a single bacterium and were often located just beneath the cell surface (Fig. 6C, panel i, arrow), whereas other vesicles were much larger and contained numerous (>50) bacteria (Fig. 6C, panel ii, arrow). In contrast, there was no evidence for internalization and replication of serotype A2 bacteria.

### Mannheimia haemolytica stimulates the release of key proinflammatory mediators by BBECs.

**FIG 7:**
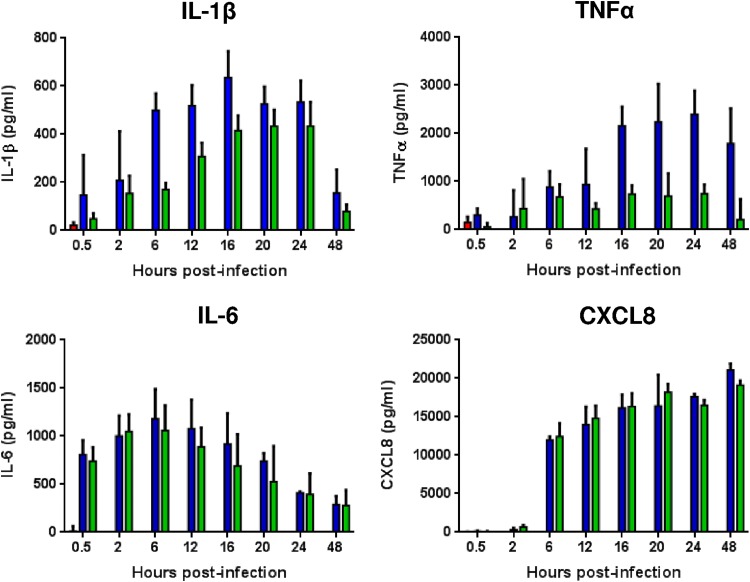
Proinflammatory innate immune response of differentiated BBECs from the basal surface following infection with M. haemolytica isolates PH2 and PH202. Differentiated BBEC cultures were infected with M. haemolytica isolates PH2 and PH202 (2.5 × 10^7^ CFU/insert) at day 21 post-ALI. At the indicated time points p.i., the levels of expression of IL-1β, TNF-α, IL-6, and CXCL8 within the basolateral medium were measured by ELISA. Cytokine/chemokine expression was quantified in two cultures at each time point, and the data represent means ± standard deviations of results determined for cultures derived from three different animals (red columns, uninfected control; blue columns, infection with isolate PH2; green columns, infection with isolate PH202).

To provide insight into the proinflammatory innate immune response of BBECs following challenge with M. haemolytica and to assess differences in the responses to serotype A1 and A2 isolates, the production of four key proinflammatory cytokines/chemokines was assessed by enzyme-linked immunosorbent assay (ELISA). Following challenge with isolate PH2 or PH202, the release of the cytokines interleukin-1β (IL-1β), interleukin-6 (IL-6), and tumor necrosis factor α (TNF-α) and of the chemokine CXCL8 was quantified at selected time points p.i. from both the basal (Fig. 7) and apical (Fig. S9) surfaces. All cytokines/chemokines exhibited a significant increase (*P ≤ *0.0001, two-way ANOVA) in production from the basal surface following infection by either PH2 or PH202 in comparison to the levels seen with uninfected controls (Fig. 7). Notably, the production of IL-1β and TNF-α was significantly higher (*P ≤ *0.0001, two-way ANOVA) in cultures challenged with serotype A1 isolate PH2 than in those challenged with serotype A2 isolate PH202, peaking at 16 and 24 h, respectively; in contrast, the IL-6 and CXCL8 responses to the two isolates were very similar. Production of IL-6 was more rapid and peaked much earlier than the production seen with the other three cytokines/chemokines at 6 h p.i. Notably, the response of the CXCL8 chemokine was delayed in comparison to that of the three cytokines; there was virtually no expression at 2 h, but a sudden increase in activity occurred at 6 h, and production continued to increase until 48 h p.i. The amount of CXCL8 produced was also generally 10-to-40-fold higher than the amounts of the three cytokines. The release of the same cytokines/chemokines from the apical surface followed the same trend as the release from the basal surface (Fig. S9).

## DISCUSSION

In the present study, we investigated the interactions of serotype A1 and A2 strains of M. haemolytica with differentiated BBECs with the aim of improving our understanding of host-pathogen interactions within the bovine respiratory tract during the early stages of BRD. Differentiated BBEC cultures recapitulate the 3-D, multicellular architecture of the *in vivo* airway epithelium; in particular, they possess the hallmark barrier functions of the bovine respiratory tract, including active mucociliary clearance and intact junctional complexes (58, 59). These mechanisms represent the first line of defense against invading pathogens and are important considerations in modeling bacterial interactions within the airways (32, 65, 70). Here, we have presented detailed BBEC infection data for single-serotype A1 and A2 isolates at frequent time points over 5 days but, importantly, our results were confirmed in further infection experiments in which we analyzed an additional isolate representing each serotype at less-frequent time points (results not shown).

The initial levels of adherence of the serotype A1 and A2 isolates to differentiated BBECs (up to 2 h p.i.) were very similar; enumeration demonstrated that adherence corresponded to approximately 1% of the inoculum. This level of adherence was in agreement with the previously observed adherence of M. haemolytica to *ex vivo* URT tissue (3% of the inoculum) (71) and BBECs (4% of the inoculum) (38) maintained under submerged conditions. Microscopy revealed that the adherence was generally sporadic, of relatively low frequency, and, notably, occurred primarily in the form of adherence to nonciliated cells rather than to ciliated cells or cilia. Interestingly, the related nontypeable Haemophilus influenzae (NTHI) also exhibits tropism toward nonciliated AECs (46), whereas other, more distantly related bacterial respiratory tract pathogens, including Moraxella catarrhalis (40), Mycoplasma pneumoniae (48, 51), Pseudomonas aeruginosa (72), and Bordetella pertussis (73), bind preferentially to ciliated AECs.

The fates of the serotype A1 and A2 isolates were very different at 6 h p.i. and later time points. Enumeration of bacteria together with immunofluorescence and electron and light microscopy data provided clear evidence that M. haemolytica serotype A1 invaded the epithelial cell layer by 12 h p.i. and rapidly replicated between 12 and 24 h p.i. to form characteristic foci of infection. The infection foci subsequently expanded by lateral spread of bacteria, which led to severe disruption and destruction of the epithelial layer by 48 to 72 h p.i. In contrast, M. haemolytica serotype A2 showed no capability or a very limited capability of infection of BBEC cultures; this strain was unable to invade and replicate within the epithelium under the described conditions. Indeed, the clearance of this isolate from BBEC cultures suggests the presence of antimicrobial activity, which is discussed further below. Evidence derived from both SEM and TEM, as well as from gentamicin protection assays, confirmed that serotype A1 M. haemolytica was internalized by BBECs and subsequently underwent rapid intracellular replication. Invasion of AECs by M. haemolytica has not previously been described and represents a new facet of disease pathogenesis and a newly discovered virulence mechanism for this bacterial species. In a previous study, submerged BBECs were infected with M. haemolytica for 3 h but invasion was not observed (38). Our findings highlight two significant advantages of using differentiated BBECs compared to submerged cultures in infection studies. First, the cultures are, by definition, differentiated and comprise the different cell types (e.g., ciliated, goblet, and basal cells) that occur in native respiratory epithelium; the cultures also possess the 3-D architecture of native epithelium and grow under physiological conditions similar to those that occur *in vivo*. Second, it is possible to perform infection experiments over long periods (up to 5 days in our case) with differentiated ALI cultures; this is not possible with submerged cultures due to bacterial growth within the culture medium. Thus, the likely explanations for the lack of observation of invasion in the previous study (38) were that the epithelial cells were undifferentiated and the incubation time was insufficient. Although invasion of AECs by M. haemolytica has not previously been described, it was not a complete surprise because this process is involved in the pathogenesis of various human respiratory tract infections caused by NTHI (46, 74–76), M. catarrhalis (61, 62) and Neisseria meningitidis (55, 60). The discovery of this invasion process led us next to question how M. haemolytica traverses the epithelium.

The airway epithelium acts as a physical barrier against infection, and microbes have evolved various strategies for crossing this barrier; these include passing between cells (paracytosis), entering and passing through cells (transcytosis), or simply killing cells to eliminate the barrier (32, 70, 77). Bacteria that cross epithelia by paracytosis typically possess mechanisms that target the tight junctions and other intercellular junctions (64, 65, 78, 79). In the present study, several lines of evidence suggested that serotype A1 M. haemolytica isolate PH2 was not traversing the epithelial layer via paracytosis by disrupting tight junctions. First, IFM revealed that bacteria were adhering to and entering cells at a central location; there was no evidence that the bacteria were associated with the cell periphery, and the lack of such an association was confirmed by SEM. Second, there was no evidence that the integrity of the epithelium, as assessed by TEER, was adversely affected during the early stages of infection such as would be expected if secreted bacterial factors were targeting tight junctions prior to invasion. The rapid decline in TEER between 16 and 48 h and the associated disruption of tight junctions were clearly due to the destruction of the epithelial layer. Third, TEM analysis identified intact tight junctions between epithelial cells whose paracellular spaces contained numerous bacteria. Fourth, the addition of LXA_4_ to BBEC cultures prior to infection increased the integrity of the tight junctions but did not reduce tissue invasion. Lipoxin A4 stimulates the expression of ZO-1, prevents tight-junction disruption, and reduces the invasion of bronchial epithelial cells by P. aeruginosa (68, 69). Thus, the addition of LXA_4_ to BBECs might be expected to reduce colonization if tight junctions were being targeted and represented the route of entry but this was not the case.

Among the major human respiratory tract pathogens, there is some evidence that NTHI traverses respiratory epithelium via paracytosis (56, 63, 80), but transcytosis plays a more prominent role in the traversal of bacterial pathogens across the respiratory epithelium. Thus, M. catarrhalis invasion occurs via macropinocytosis involving microfilaments and the formation of lamellipodia (61, 62) and N. meningitidis traverses the respiratory epithelial barrier via an intracellular microtubule-dependent route (55, 60). Micropinocytosis involving the formation of microvilli and lamellipodia (46), receptor-mediated endocytosis (81, 82), and lipid raft-independent endocytosis (74) have been cited as mechanisms for the internalization of NTHI by AECs. Assessing the precise mechanism of BBEC invasion by M. haemolytica was beyond the scope of the present study, but SEM and TEM analyses of infected BBECs failed to identify any evidence of the membrane ruffling and lamellipodium formation that are characteristic of NTHI (46, 83) and M. catarrhalis (61) interactions with human AECs. However, SEM imaging did reveal evidence for the potential uptake of M. haemolytica by nonciliated epithelial cells in the form of large numbers of bacteria associated with membrane invaginations. These invaginations may be linked with mucus extrusion, and it is interesting to speculate that goblet cells are perhaps involved in bacterial uptake, but further evidence is required to confirm this. A role for goblet cells in M. haemolytica invasion would not be entirely surprising because Listeria monocytogenes specifically targets this cell type to gain entry to the intestinal epithelium (84). Although the precise mechanism involved remains to be elucidated, we propose that transcytosis rather than paracytosis represents the most likely route of epithelial invasion by serotype A1 M. haemolytica.

TEM analysis demonstrated that, after internalization, serotype A1 isolate PH2 became enclosed within membrane-bound vacuoles or endosomes. The presence of large numbers of bacteria within these vacuoles, together with the very rapid increase in bacterial numbers associated with the BBEC cultures between 12 and 24 h, suggests that M. haemolytica serotype A1 is capable of very rapid intracellular replication after internalization. Thus, AECs appear to provide a suitable microenvironment for which M. haemolytica is adapted. The ability to invade, survive, and replicate within AECs provides M. haemolytica with a potential intracellular niche shielding the bacterium from antibodies, complement, and antibiotics and potentially allowing persistence for extended periods of time (74, 75, 80). Although persistence within AECs has not previously been considered as a possible survival strategy for M. haemolytica, our findings nonetheless raise the possibility that bacteria internalized within AECs could act as a reservoir of infection (74, 75, 85), potentially leading to recurrent or reemergent colonization of the URT (17).

**FIG 8:**
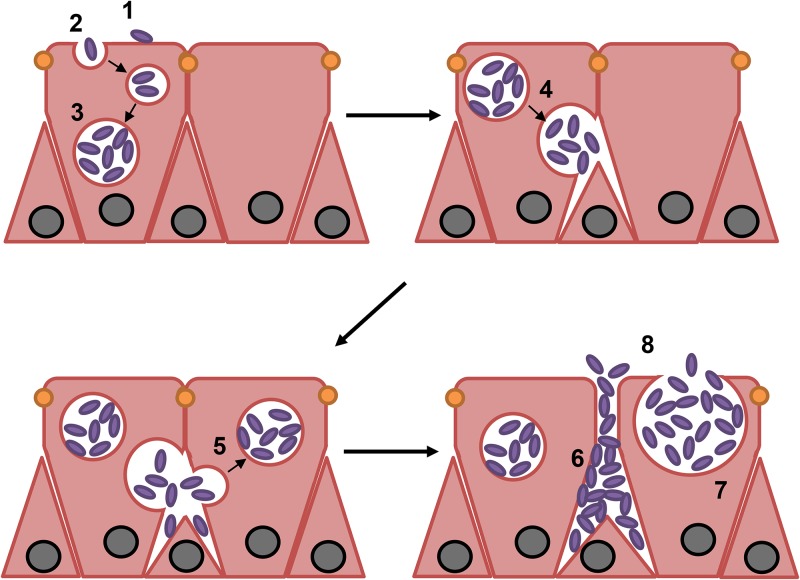
Proposed model for the internalization and infection of differentiated BBECs by serotype A1 M. haemolytica. Bacteria adhere to the apical surface of nonciliated epithelial cells (step 1) and are taken up via an endocytosis-type mechanism (step 2). Rapid replication of internalized bacteria occurs within endosomes (step 3), and these fuse with the lateral membranes, releasing bacteria into the paracellular spaces (step 4). Bacteria may gain entry into adjacent cells (step 5), and further replication within the paracellular spaces (step 6) and cytoplasm (step 7) leads to the development of infection foci, disruption of tight junctions, and rupture and death of epithelial cells and to the release of large numbers of bacteria onto the epithelial cell surface (step 8).

On the basis of the observations described above, we propose a model for the invasion of differentiated BBECs by serotype A1 M. haemolytica (Fig. 8). After initial adherence to the epithelial cell surface (i), bacteria are taken up by nonciliated epithelial cells via an endocytotic-type mechanism (ii). Rapid bacterial replication (iii) subsequently occurs within endosomes present in the cytoplasm of infected epithelial cells, and these likely fuse with the lateral membranes releasing bacteria into the paracellular spaces (iv). Bacteria are taken up laterally by adjacent epithelial cells (v), and further replication within the paracellular spaces (vi) and in secondarily infected cells (vii) occurs. These events very quickly lead to disruption of tight junctions, rupture and death of epithelial cells, and the release of large numbers of bacteria onto the epithelial surface (viii). Thus, the initial uptake of a relatively small number of bacteria leads to their rapid replication and spread to adjacent cells, resulting in the observed formation of foci of infection and destruction of the epithelial cell layer. The rapid replication and release of large numbers of bacteria onto the epithelial surface, as proposed in this model, provide a potential mechanism which might explain the explosive proliferation of serotype A1 M. haemolytica that occurs in the URT of cattle prior to the onset of disease. Although *in vivo* evidence for invasion of respiratory airway epithelium by M. haemolytica is lacking, it is noteworthy that descriptions of the pathology and histopathology of BRD focus almost exclusively on lesions of the lungs (2, 17, 24, 86, 87). In addition, experimental challenge studies involving M. haemolytica typically involve intratracheal inoculation of bacteria and subsequent assessment that again focuses on changes in lung pathology (13, 88–92). This challenge method does not replicate natural infection, and host-pathogen interactions involving airway epithelia of the trachea and upper respiratory tract are completely bypassed.

In contrast to PH2, serotype A2 isolate PH202 was unable to colonize differentiated BBECs and, by day 5 pi, was cleared from cultures derived from two of the three animals. These observations suggest that PH202 was susceptible to the antibacterial activity of AECs. An important function of airway epithelia is the production of antimicrobial peptides, including defensins and cathelicidins, in response to infection (32, 77, 93). Indeed, it is becoming increasingly clear that the production of antimicrobial peptides, such as tracheal antimicrobial peptide, by ruminant respiratory epithelial cells plays an important role in combating BRD (22, 94–97). Thus, our observations highlight further potential differences between serotype A1 and A2 isolates of M. haemolytica in terms of their responses to exposure to the antimicrobial activity of bovine AECs.

In addition to their barrier function, AECs also play an important role in orchestrating the host innate immune response to infection through the release of proinflammatory cytokines and chemokines (33, 77, 98, 99). The proinflammatory cytokines IL-1β, IL-6, and TNFα as well as the proinflammatory chemokine CXCL8 are produced by bovine AECs during BRD (23, 34, 35, 100). In the present study, challenge of differentiated BBECs stimulated the release of IL-1β, IL-6, TNFα, and CXCL8 by strains of both M. haemolytica serotypes. Notably, serotype A1 isolate PH2 resulted in significantly higher levels of IL-1β and TNF-α than did serotype A2 strain PH202. Insights into the kinetics of proinflammatory mediator release were also gained. Thus, production of IL-6 occurred very rapidly and peaked at 6 h whereas production of the other cytokines/chemokines, especially CXCL8, occurred more slowly and peaked later. There were also quantitative differences in the levels of cytokine/chemokine production: in particular, production of CXCL8 was 10-fold to 40-fold higher than the levels measured for the three cytokines. Importantly, the earlier production of IL-1β and TNF-α than of CXCL8 and the higher levels of CXCL8 production agree with previously reported kinetic profile and quantitative data on cytokine/chemokine production in the airways of calves infected with M. haemolytica (23). Thus, the proinflammatory innate immune response of differentiated BBECs challenged with M. haemolytica closely mimics the *in vivo* response of bovine AECs in infected calves and provides partial validation for use of the model in such studies.

In summary, we have demonstrated that serotype A1 M. haemolytica but not serotype A2 M. haemolytica invades differentiated AECs and subsequently undergoes rapid intracellular replication before spreading to adjacent cells and causing extensive cellular damage. The differing abilities of serotype A1 and serotype A2 M. haemolytica isolates to invade and damage the airway epithelium correlate with the behavior of these strains *in vivo* and support the idea of the relevance of using differentiated BBECs for studying the pathogenesis of M. haemolytica disease. In particular, our findings may provide insight into the previously unexplained and sudden explosive proliferation of serotype A1 bacteria that occurs within the bovine respiratory tract prior to the onset of pneumonic disease. The identification of an invasion mechanism in serotype A1 M. haemolytica but not serotype A2 M. haemolytica represents a significant step forward in understanding why the former, and not the latter, is responsible for the majority of disease outbreaks. Our findings suggest that serotype A1 strains possess previously unrecognized virulence determinants associated with invasion that may represent potential new vaccine and/or drug targets. Understanding the molecular basis of AEC invasion may provide opportunities for the development of new and improved prevention and treatment strategies that target early colonization of the bovine URT. Finally, we have demonstrated that differentiated BBECs are excellent mimics of the bovine respiratory epithelium and represent a realistic and potentially powerful *in vitro* tool for studying the interactions of M. haemolytica and other BRD-associated pathogens with their bovine host. Thus, the BBEC infection model described here has broad applications and significant potential for replacing or reducing the use of cattle in BRD research.

## MATERIALS AND METHODS

### Bacterial strains and growth conditions.

Two key M. haemolytica reference strains, PH2 and PH202, were used in this study. Isolate PH2 is of serotype A1 and was isolated from the lungs of a confirmed case of bovine pneumonic pasteurellosis, whereas isolate PH202 is of serotype A2 and was recovered from the nasopharynx of a clinically healthy calf on a disease-free farm. The two isolates were characterized in previous comparative studies of M. haemolytica (27, 28, 31, 101–103). The bacterial isolates were stored at −80°C in 50% (vol/vol) glycerol–brain heart infusion broth (BHIB; Oxoid) and were subcultured on brain heart infusion agar (Oxoid) containing 5% (vol/vol) defibrinated sheep’s blood (blood agar) overnight at 37°C. Broth cultures were prepared by inoculation of 25-ml volumes of BHIB from overnight growth on blood agar and incubation at 37°C and 120 rpm.

### Isolation and culture of differentiated BBECs.

Differentiated BBECs were prepared from primary bronchial epithelial cells recovered from the lungs of freshly slaughtered, 24-to-30-month-old cattle as described previously (58). Briefly, the lungs were transported to the laboratory on ice and the left and right bronchi were dissected and sections incubated overnight at 4°C in digestion medium (DM). Epithelial cells were recovered from the bronchial sections and resuspended in submerged growth medium (SGM) at a cell density of 5.0 × 10^5^ cells/ml. Volumes of cell suspension (10 ml) were seeded into T75 tissue culture flasks and incubated at 37°C in a humidified atmosphere containing 5% CO_2_ and 14% O_2_. The BBECs were grown until 80% to 90% confluent (∼4 days), subjected to trypsinization, and resuspended in SGM to a density of 5.0 × 10^5^ cells/ml. Subsequently, 0.5 ml of the cell suspension was seeded onto the apical surface of 12-mm-diameter, PET ThinCert membrane inserts of 0.4-μm pore diameter and containing 1.0 × 10^8^ pores per cm^2^ (Greiner; catalog no. 665640). The epithelial cells were cultured at 37°C in a humidified atmosphere containing 5% CO_2_ and 14% O_2_ and were fed every 2 to 3 days. The TEER of the cultures was measured daily using an EVOM2 epithelial volt-ohm-meter (World Precision Instruments, United Kingdom) according to the manufacturer’s instructions. When the TEER reached 200 Ω/cm^2^ or above (∼2 days), the growth medium was replaced with a 50:50 mixture of SGM and air-liquid interface (ALI) medium (containing 10 ng/ml epidermal growth factor and 100 nM retinoic acid). When the TEER reached 500 Ω/cm^2^ (indicating successful barrier formation), an ALI was generated (this represented day 0 post-ALI) and the cells were fed exclusively from the basal compartment with ALI medium every 2 to 3 days until a well-differentiated epithelial layer was obtained (see Fig. S10 in the supplemental material).

### Infection of bovine bronchial epithelial cells.

Differentiated BBEC cultures were infected on day 21 post-ALI (59). At 24 h prior to infection, the basal medium was removed, the apical and basal compartments were washed twice with phosphate-buffered saline (PBS), and the basal compartment was replenished with 1.0 ml of antibiotic-free ALI medium. On the day of infection, bacterial broth cultures were grown to exponential phase (4 to 5 h), and the bacteria were harvested by centrifugation, washed, and resuspended in PBS to a cell density of 1.0 × 10^9^ CFU/ml. The apical surface of each BBEC culture was washed with 0.5 ml PBS and inoculated with 25 μl of bacterial suspension (2.5 × 10^7^ CFU/insertion), and the infected cultures were incubated at 37°C in a humidified atmosphere containing 5% CO_2_ and 14% O_2_. The BBEC cultures were assessed at 0.5, 2, 6, 12, 16, 20, 24, 48, 72, and 120 h p.i.

### Quantification of bacterial adherence and colonization.

Bacterial enumeration was performed at each time point following infection by performing counts of viable cells. The ALI medium was removed from the basal compartment, and the apical surface of each infected BBEC culture was washed three times with 1 ml PBS to remove unattached bacteria. The volumes of washes were pooled, and the number of viable bacteria present was determined as described below. The BBEC layer was disrupted by the addition of 0.5 ml of 1% Triton X-100–PBS to the apical surface for 10 min followed by mechanical scraping and pipetting. Numbers of viable bacteria in both the apical washes and BBEC lysates were quantified, in triplicate, using a method of bacterium counting described previously by Miles et al. (104). Bacterial numbers were expressed as a percentage of the inoculum. In some experiments, intracellular bacteria were enumerated using the gentamicin protection assay. Under those conditions, the apical surface of the epithelial layer was incubated with 0.5 ml of gentamicin (200 μg/ml) for 1 h at 37°C prior to disruption and counting performed as described above. Bacterial enumeration was performed in three independent BBEC cultures at each time point and using cells from three different animals (*n* = 9).

### Histology and immunohistochemistry.

Infected BBEC cultures representing each time point p.i. were fixed, processed, and sectioned as previously described (58), and the sections were subjected to either H&E staining or IHC staining. In the latter case, bacteria were identified by incubation for 30 min with a 1:800 dilution of rabbit anti-OmpA antibody (103), application of an anti-rabbit horseradish peroxidase (HRP)-labeled polymer, and visualization with a Real EnVision peroxidase/DAB^+^ detection system (Dako; catalog no. K3468); samples were subsequently counterstained with Gill’s hematoxylin. Tissue sections were viewed with a Leica DM2000 light microscope.

### Immunofluorescence microscopy.

Infected BBEC cultures representing each time point p.i. were fixed and processed for IFM as previously described (58). Tight-junction formation and cilia were detected with anti-ZO-1 and anti-β-tubulin antibodies, respectively. Bacteria were detected with a 1:50 dilution of bovine anti-M. haemolytica whole-cell antibodies and visualized with goat anti-bovine-fluorescein isothiocyanate (FITC) antibodies used at a dilution of 1:400 (Thermo Fisher; catalog no. A18752). Standard IFM images were acquired with a Leica DMi8 microscope. Z-stack orthological representation was performed on a Zeiss AxioObserver Z1 spinning-disk confocal microscope. Analysis of captured images was performed using ImageJ software.

### Scanning electron microscopy.

Infected BBEC cultures representing each time point p.i. were fixed and processed for SEM as previously described (58). The cultures were analyzed with a JEOL 6400 scanning electron microscope at 10 kV.

### Transmission electron microscopy.

Infected BBEC cultures representing selected time points p.i. were fixed and processed for TEM as previously described (59). The cultures were analyzed on a FEI Tecnai transmission electron microscope at 200 kV and images captured with a Gatan Multiscan 794 camera.

### Proinflammatory cytokine/chemokine analysis.

Production of IL-1β, IL-6, TNF-α, and CXCL8 (IL-8) from both the apical and basal surfaces of infected and uninfected (PBS alone added to the apical surface) BBEC cultures was assessed at each time point p.i. To measure cytokine/chemokine production from the basal surface, 1 ml of medium was removed from the basolateral compartment and centrifuged at 5,000 × *g* for 5 min, and the supernatant was immediately frozen at −80°C. To measure levels of cytokine/chemokine production from the apical surface, 0.5 ml of antibiotic-free ALI medium was added to the apical surface of each culture and the cultures were returned to the incubator for 30 min. The medium was subsequently removed and centrifuged at 5,000 × *g* for 5 min, and the supernatant was immediately frozen at −80°C. Cytokine/chemokine production was quantified using commercially available enzyme-linked immunosorbent assay (ELISA) kits according to the instructions of the manufacturers as follows: IL-1β, bovine IL-1β ELISA reagent kit (Thermo Fisher; catalog no. ESS0027); IL-6, bovine IL-6 ELISA reagent kit (Thermo Fisher; catalog no. ESS0029); CXCL8, bovine IL-8 ELISA development kit (Mabtech; catalog no. 3114-1A-6); TNF-α, bovine TNF-α DuoSet ELISA development system (R&D systems; catalog no. DY2279). Triplicate samples were measured for each insertion, and two individual cultures were analyzed for each donor animal (*n* = 6).

### Data analysis.

Unless otherwise stated, all experiments were independently performed three times using epithelial cells derived from three individual donor animals and, for quantitative analysis, three separate cultures from each donor animal were analyzed (*n* = 9). Results are presented as means ± standard deviations. Data were statistically analyzed using one-way or two-way ANOVA for comparisons of one or two independent variables, respectively. Significance was indicated by a *P* value of less than 0.05. Analyses were performed using GraphPad Prism (GraphPad Software Inc.).

## Supplementary Material

Supplemental file 1
