# Supplementary material for: Pathogenic Mannheimia haemolytica Invades Differentiated Bovine Airway Epithelial Cells
Source: Infect Immun. 2019 May 21;87(6):e00078-19. doi: 10.1128/IAI.00078-19 (PMC6529648; doi:10.1128/IAI.00078-19)
Supplement: Supplemental file 1 [file IAI.00078-19-s0001.pdf]

Supplemental Material

Pathogenic *Mannheimia haemolytica* invades differentiated bovine airway epithelial cells

Daniel Cozens[1], Erin Sutherland[1], Miquel Lauder[1], Geraldine Taylor[2], Catherine C. Berry[3] and Robert L. Davies[1#]

[1] *Institute of Infection, Immunity and Inflammation, College of Medical, Veterinary and Life Sciences, University of Glasgow, Glasgow, UK*
[2] *The Pirbright Institute, Pirbright, Surrey, UK*
[3] *Institute of Molecular, Cell and Systems Biology, College of Medical, Veterinary and Life Sciences, University of Glasgow, Glasgow, UK*

[#]Corresponding author e-mail: robert.davies@glasgow.ac.uk

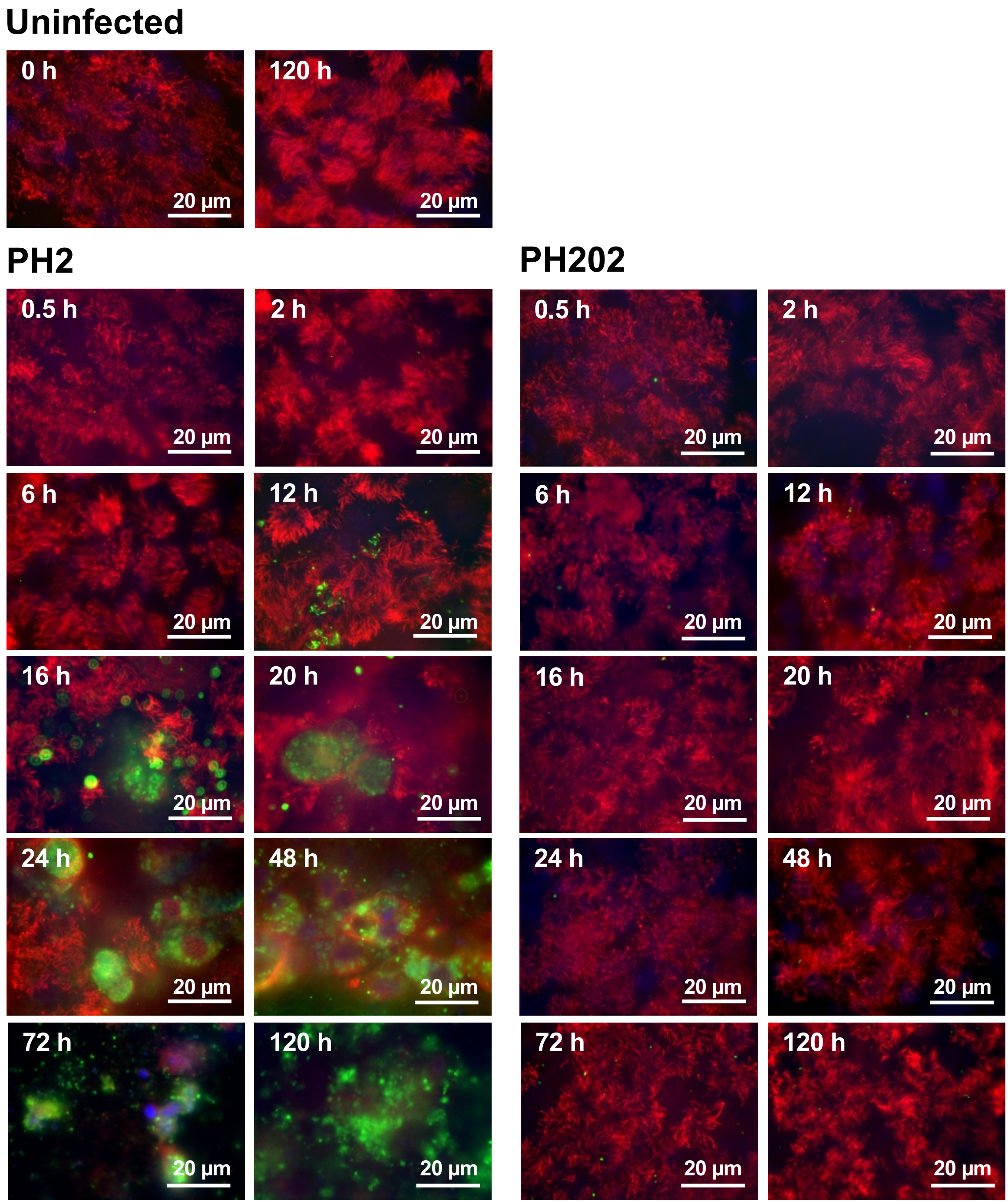

FIG S1 Dynamics of PH2 and PH202 infection of differentiated BBECs over 5-day time-course using IFM. Differentiated BBEC cultures were infected with *M. haemolytica* isolates PH2 and PH202 (2.5 x 10^7 cfu/insert) at day 21 post-ALI and maintained for five days. At the indicated time points pi, the cultures were washed to remove unbound bacteria, fixed and bacterial colonisation assessed using IFM (bacteria - green; cilia [β-tubulin] - red; nuclei - blue). Increasing numbers of PH2 but not PH202 bacteria were associated with BBECs from 12 h pi and PH2 infection caused increasing damage to the epithelial layer after 24 h.

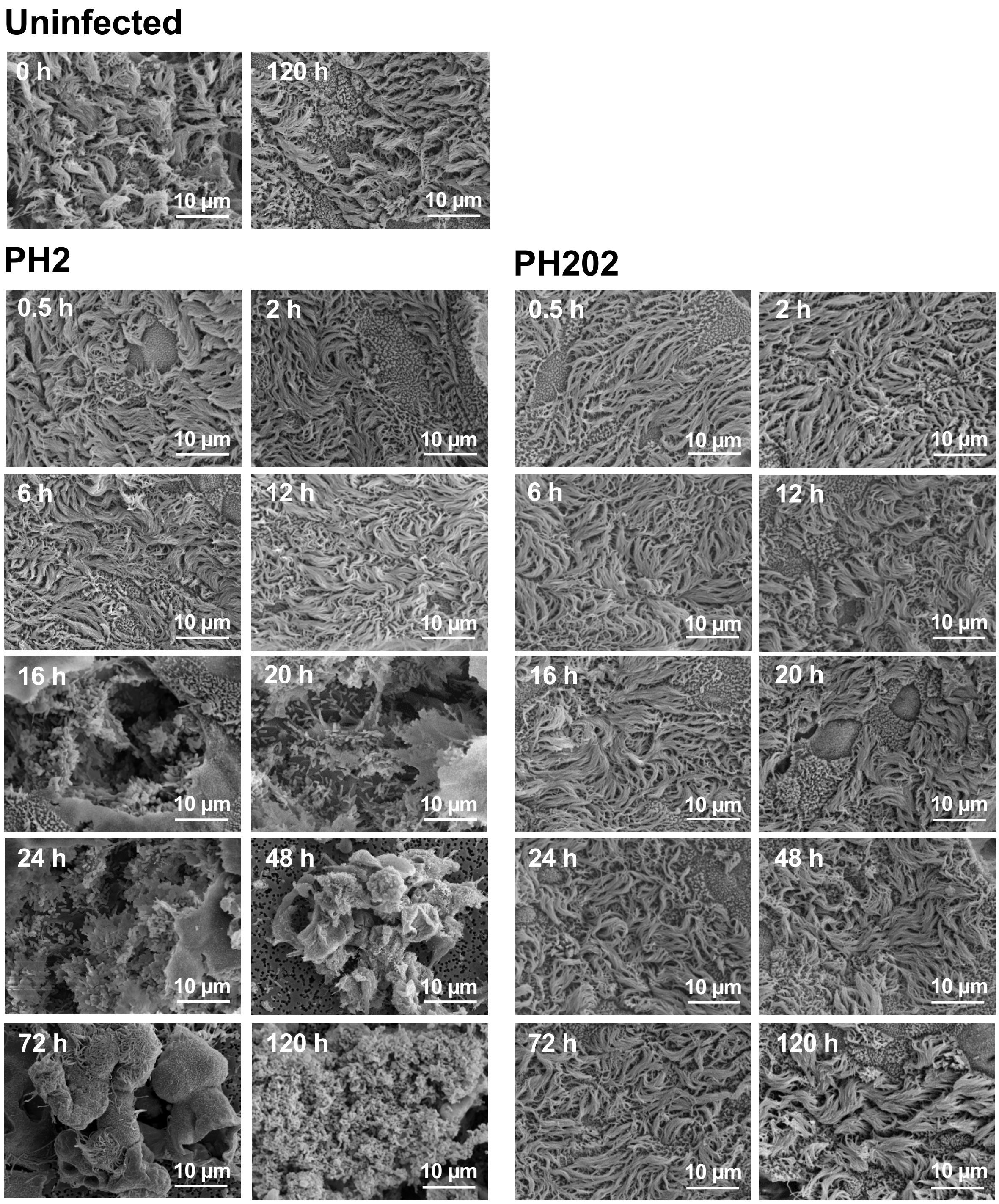

FIG S2 Dynamics of PH2 and PH202 infection of differentiated BBECs over 5-day time-course infection using SEM. Differentiated BBEC cultures were infected with *M. haemolytica* isolates PH2 and PH202 (2.5 x 10^7 cfu/insert) at day 21 post-ALI and maintained for five days. At the indicated time points pi, the cultures were washed to remove unbound bacteria, fixed and bacterial colonisation assessed using SEM. Increasing numbers of PH2 but not PH202 bacteria were associated with damaged tissue from 16 h pi and PH2 infection caused increasing damage to the epithelial layer after 24 h.

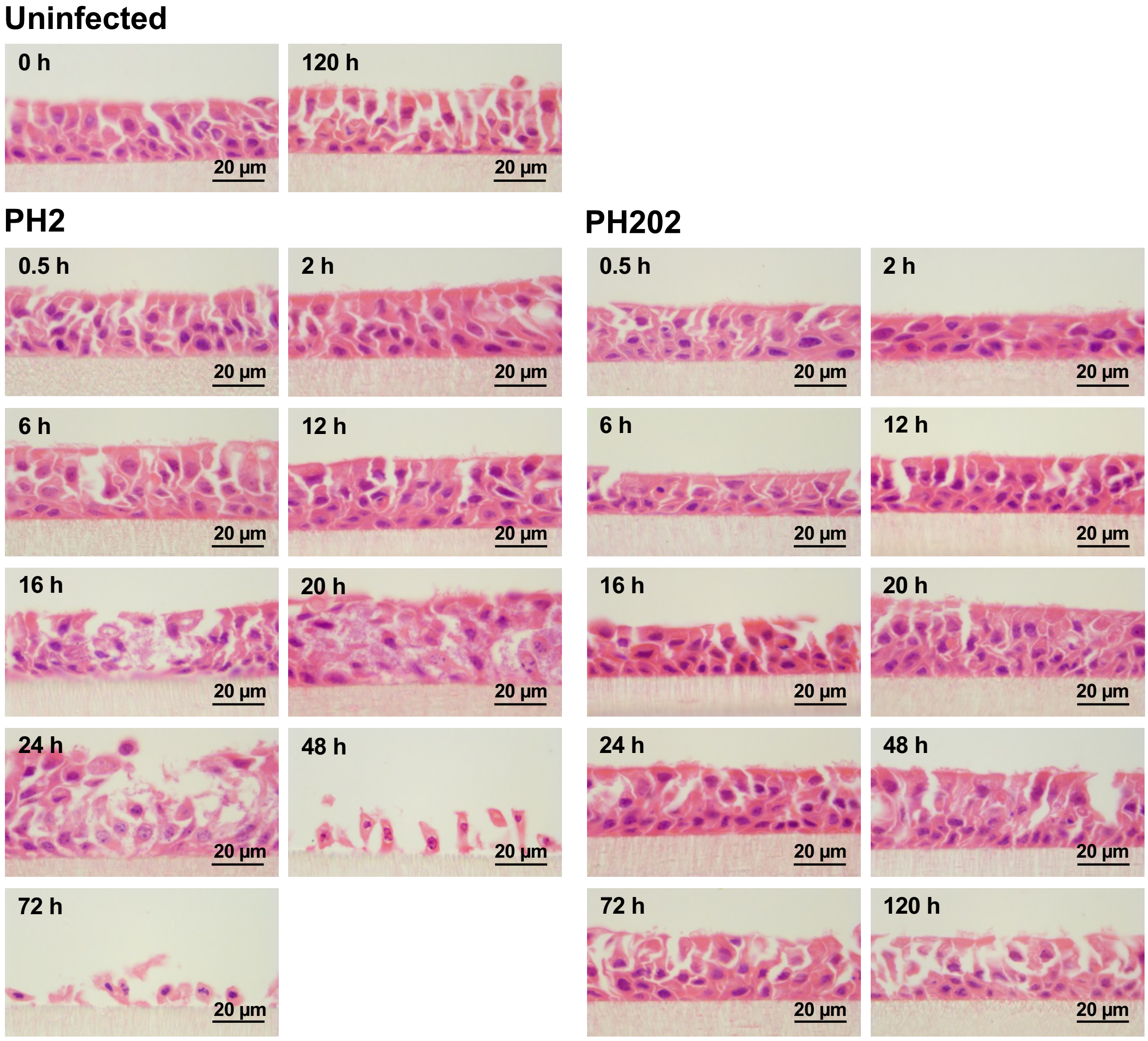

FIG S3 Dynamics of PH2 and PH202 infection of differentiated BBECs over 5-day time-course using histological analysis. Differentiated BBEC cultures were infected with *M. haemolytica* isolates PH2 and PH202 (2.5 x 10^7 cfu/insert) at day 21 post-ALI and maintained for five days. At the indicated time points pi, the cultures were washed to remove unbound bacteria, fixed, paraffin-embedded, sectioned and stained with H&E using standard histological techniques. Increasing numbers of isolate PH2 but not PH202 bacteria were observed within the epithelial layer from 16 h pi, apoptotic and rounded cells were present at 24 h and severe damage to the epithelial layer occurred from 48 h. Note: the 120 h sample for PH2 was too damaged to allow sectioning.

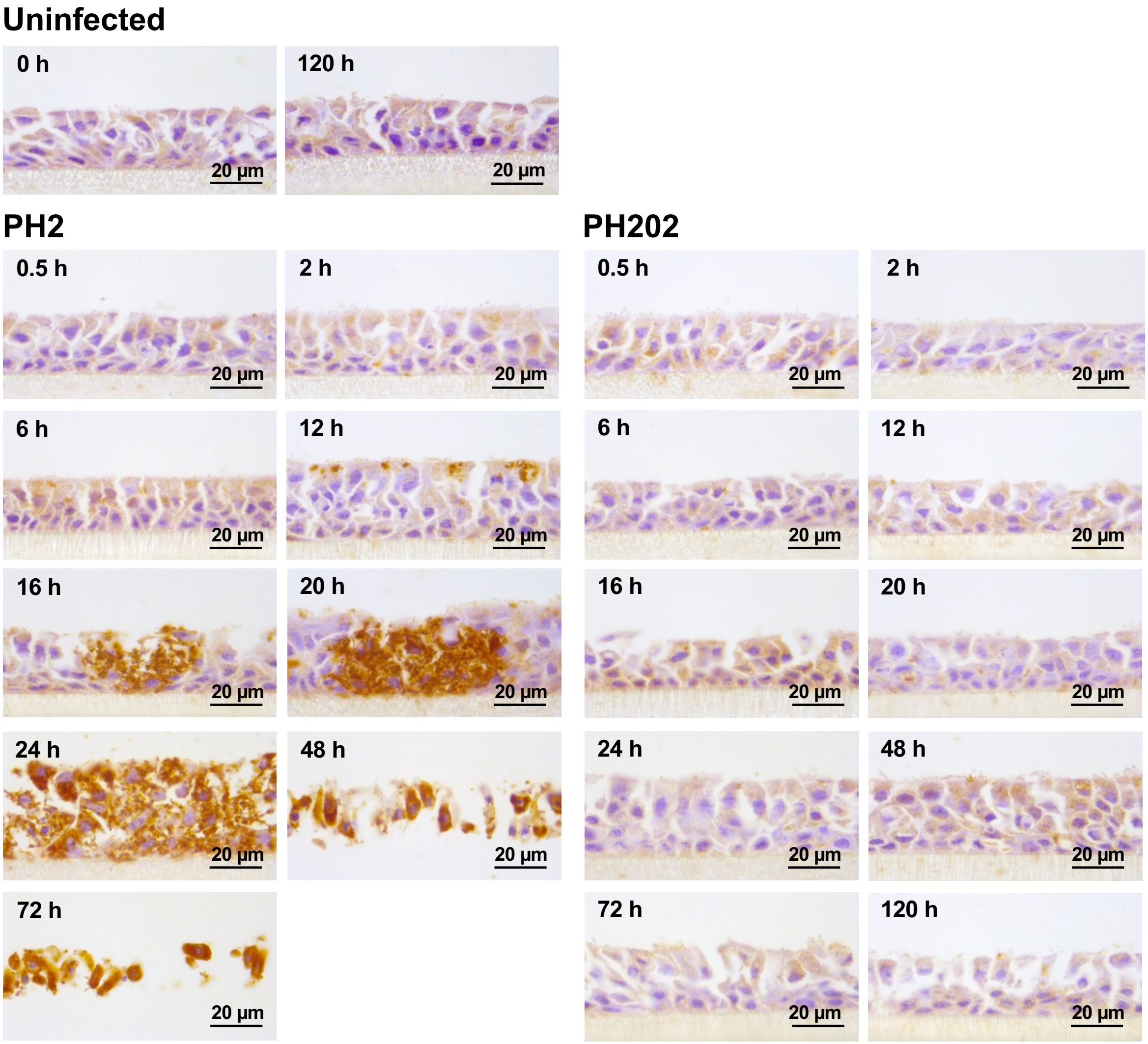

FIG S4 Dynamics of PH2 and PH202 infection of differentiated BBECs over 5-day time-course using immunohistochemical analysis. Differentiated BBEC cultures were infected with *M. haemolytica* isolates PH2 and PH202 (2.5 x 10^7 cfu/insert) at day 21 post-ALI and maintained for five days. At the indicated time points pi, the cultures were washed to remove unbound bacteria, fixed, paraffin-embedded, sectioned and subjected to IHC-staining (OmpA-labelled bacteria are stained brown). Isolate PH2 but not PH202 bacteria were identified within the epithelial layer from 12 h pi and distinct foci of infection, penetrating the full depth of the epithelial layer, were visible by 16 h. Infection foci had increased in size and number by 20 h and bacteria had spread extensively throughout the epithelial layer by 24 h. Extensive damage to the epithelial layer was apparent from 48 h. Note: the 120 h sample for PH2 was too damaged to allow sectioning.

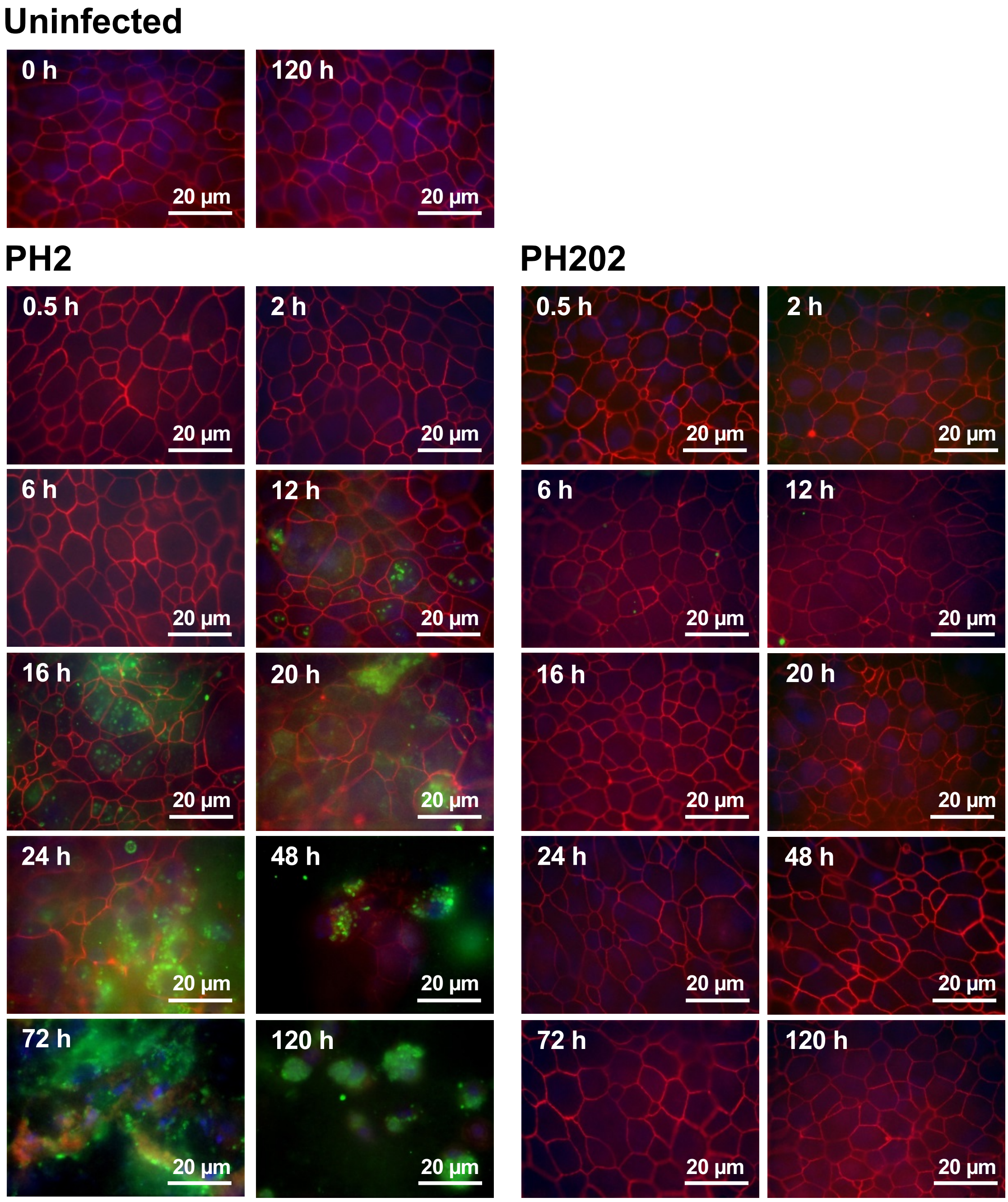

FIG S5 Tight junction integrity of differentiated BBEC cultures infected with *M. haemolytica* isolates PH2 and PH202 over 5-day time-course. Differentiated BBEC cultures were infected with *M. haemolytica* isolates PH2 and PH202 (2.5 x 10^7 cfu/insert) at day 21 post-ALI and maintained for five days. At the indicated time points pi, the cultures were washed to remove unbound bacteria and fixed. Bacterial colonisation and tight junction integrity were subsequently assessed using IFM (bacteria - green; ZO-1 - red; nuclei - blue). Increasing numbers of PH2 bacteria were associated with BBECs over time and tight-junctions remained intact until 16 h, after which localised disruption was observed at 20 and 24 h, followed by complete disruption from 48 h which was associated with severe damage to the epithelial cells. In contrast, PH202 bacteria did not colonise the BBECs and tight junctions remained intact throughout the duration of the 5-day time-course.

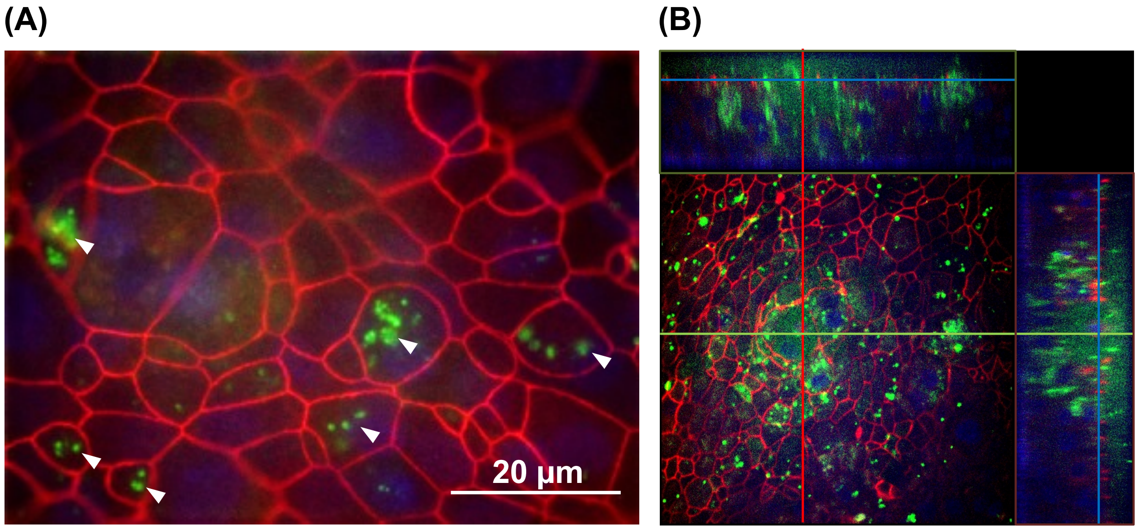

FIG S6 Infection by isolate PH2 at early time-points does not affect epithelial cell tight junction integrity and has a central cellular location. Differentiated BBEC cultures infected with *M. haemolytica* isolate PH2 were assessed by (A) IFM at 12 h pi and (B) confocal microscopy at 24 h pi (bacteria - green; ZO-1 - red; nuclei - blue). In (A), intact tight junctions are present in epithelial cells with which bacteria (arrowheads) are associated in a central location (note: no bacteria are associated with the cell peripheries). In (B), a Z-stack orthogonal representation (630x magnification) shows that tight junctions are intact in areas adjacent to infected cells and despite invasion of the epithelial layer.

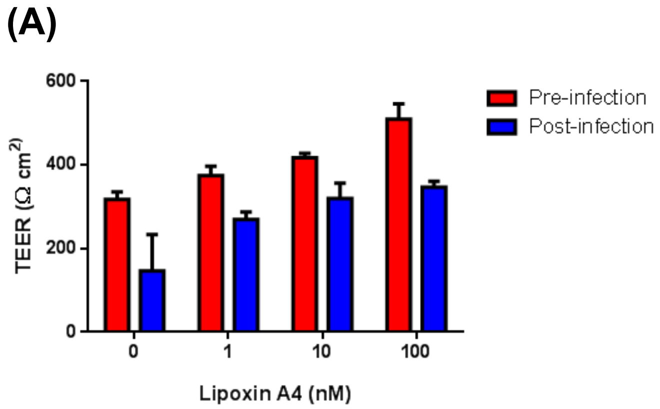

FIG S7 Lipoxin A_4 increases tight junction integrity but has no effect on bacterial invasion. Differentiated BBEC cultures were treated for 18 h with increasing concentrations of lipoxin A_4 (LXA_4) prior to infection with *M. haemolytica* isolate PH2 (2.5 x 10^7 cfu/insert) for 24 h after which bacterial numbers were determined. Tight-junction integrity of the BBEC cultures was assessed by measuring TEER pre- and post-infection with *M. haemolytica*. (A) shows that LXA_4 increases tight junction integrity of BBECs in a dose-dependent manner both pre- and post-infection; (B) shows that LXA_4 does not influence infection by isolate PH2 after 24 h - it has no effect on the numbers of bacteria colonising the cultures. For all assays, three inserts were analysed per condition and the data represent the mean +/- standard deviation.

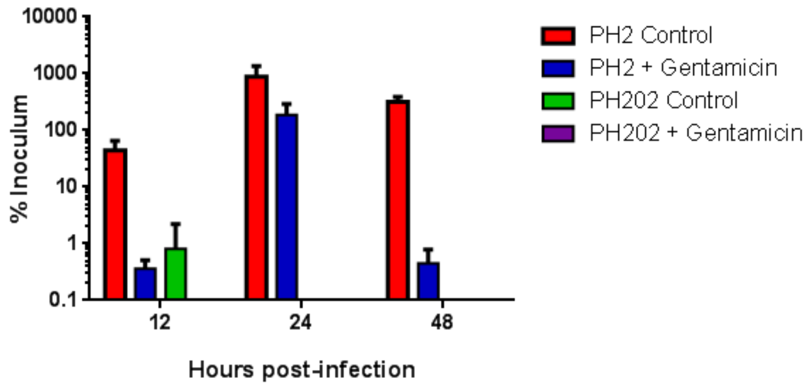

FIG S8 Gentamicin protection assay demonstrates intracellular location of isolate PH2. Differentiated BBEC cultures were infected with *M. haemolytica* isolates PH2 and PH202 (2.5 x 10^7 cfu/insert) at day 21 post-ALI. At 12, 24 and 48 h pi, gentamicin was added to the cultures for 60 min. The cultures were subsequently washed to remove unbound bacteria, the BBECs were lysed and the numbers of intracellular bacteria (expressed as a percentage of the inoculum) determined by viable counts. Isolate PH2 was present intracellularly at all time points (peaking at 24 h) whereas isolate PH202 was not detected at any time points in the presence of gentamicin. Three inserts were analysed per condition and the data represent the mean +/- standard deviation.

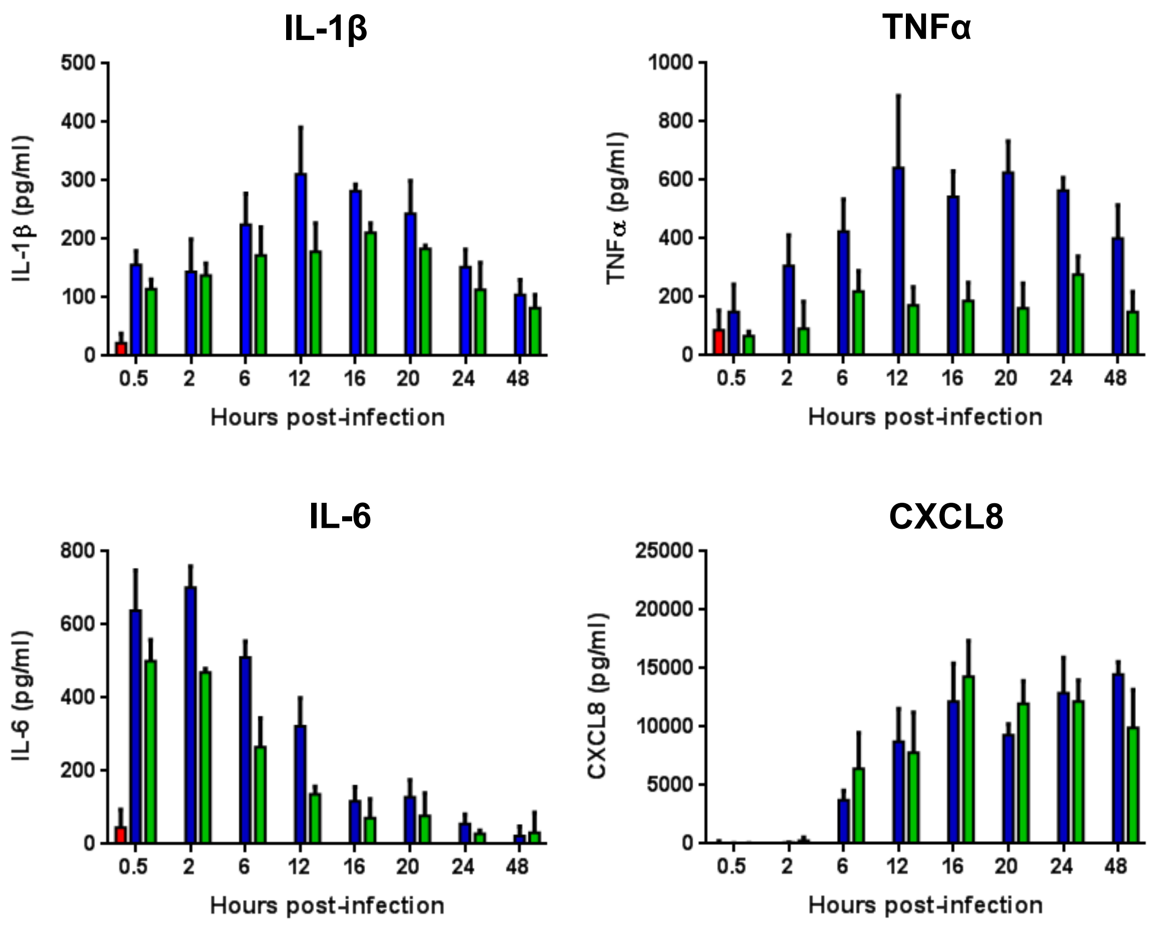

FIG S9 Proinflammatory innate immune response of differentiated BBECs from the apical surface following infection with *M. haemolytica* isolates PH2 and PH202. Differentiated BBEC cultures were infected with *M. haemolytica* isolates PH2 and PH202 (2.5 x 10^7 cfu/insert) at day 21 post-ALI. At the indicated time points pi, the expression of IL-1β, TNFα, IL-6 and CXCL8 onto the epithelial surface was measured in apical washes by ELISA. Cytokine/chemokine expression was quantified in two inserts at each time point, and the data represent the mean +/- standard deviation of cultures derived from three different animals (■, uninfected control; ■, infection with isolate PH2; ■, infection with isolate PH202.

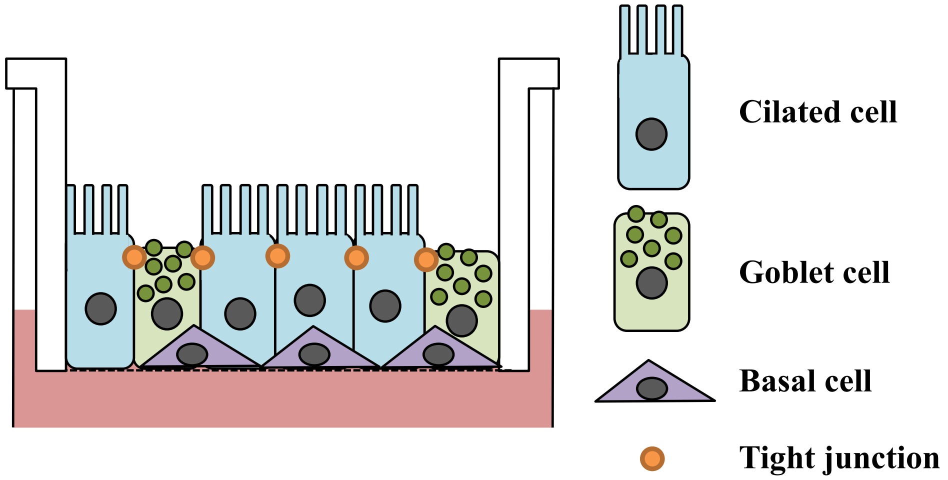

FIG S10 Schematic representation of differentiated BBECs growing at an ALI. Individual ciliated, goblet and basal cells are shown as identified previously (58, 59).
